# Design, Develop, and Pilot-Test a Digital Platform to Enhance Student Well-Being: Protocol for a Mixed-Methods Study

**DOI:** 10.2196/39779

**Published:** 2024-07-23

**Authors:** Ashish Joshi, Kamalpreet Kaur, Ashruti Bhatt, Krishna Mohan Surapaneni, Ashoo Grover, Apurva Kumar Pandya

**Affiliations:** 1 School of Public Health University of Memphis Memphis, TN United States; 2 Foundation of Healthcare Technologies Society New Delhi India; 3 Panimalar Medical College Hospital & Research Institute Chennai India; 4 Indian Council of Medical Research New Delhi India; 5 Parul Institute of Public Health Vadodara, Gujarat India

**Keywords:** well-being, students, digital interventions, social support, family demands, mental well-being

## Abstract

**Background:**

Well-being is a multidimensional concept and has been extended to many areas. Student well-being has garnered attention over the last decade due to concerns that have been raised. Digital health interventions have the potential to enhance and improve student well-being.

**Objective:**

The objective of the study is to design, develop, and pilot-test a digital health platform to enhance student well-being.

**Methods:**

A sample size of 5000 participants will be recruited across Gujarat and Tamil Nadu, India. Students will be enrolled from Parul University in Vadodara, Gujarat, as well as Panimalar Medical College Hospital and Research Institute, Panimalar Engineering College, Panimalar Institute of Technology, and Panimalar College of Nursing in Chennai, Tamil Nadu. Current undergraduate and graduate students consenting to participate will be recruited using convenience sampling from these institutes. The study will collect baseline data to construct the student well-being index. Based on the risk profile, a random subset of the population will be provided access to the digital health intervention, which will deliver tailored interactive messages addressing the various dimensions of well-being among undergraduate and graduate students. The eligible study participants will be aged 18 years and older, enrolled in these institutes, and willing to give their consent to participate in the study.

**Results:**

The proposed research is an unfunded study. The enrollment of the individuals in the study began in October 2022. Data gathered will be analyzed using SAS (version 9.3; SAS Institute) and results will be reported as 95% CIs and *P* values.

**Conclusions:**

The proposed study will help to determine the factors affecting well-being among college students and help in designing digital health interventions to improve the well-being of undergraduate and graduate students.

**International Registered Report Identifier (IRRID):**

PRR1-10.2196/39779

## Introduction

### Background and Rationale

Well-being is a multifaceted concept that encompasses 3 dimensions: mental, social, and physical [1]. According to the World Health Organization, “Health is a state of complete physical, mental and social well-being and not merely the absence of disease or infirmity” [2]. Therefore, well-being can be summarized as a positive outcome that constitutes physical, mental, and social stability. It comprises the presence of positive emotions such as happiness and resilience and the absence of negative emotions such as anxiety and depression [3].

Over the last decade, there has been an increased interest in student well-being [4]. The transitional period from school to university puts forward many new challenges and changes for students, such as academic demands, facing new people, making independent choices, and being away from home and social networks. These shifts or changes act as potent stressors in the lives of students [5].

In a survey conducted by the World Health Organization in 21 countries to assess the associations of mental disorders with college entry and attrition, it was found that anxiety disorders were the most prevalent class of disorders, followed by mood disorders, substance disorders, and behavioral disorders [6]. Another cross-sectional study conducted across 30 low- and middle-income countries drew similar conclusions [7]. A study of undergraduate students at a South African university reported minimal to mild symptoms of depression in 87.7% of students and moderate to severe symptoms in 11.2% of students. Similarly, low anxiety was prevalent in 84.2% of students, and moderate to severe anxiety was present in 5.8% of students [8]. Additional studies in Nigeria and Kenya also reported that moderate to severe depression was prevalent among university students [9,10].

For medical education students, the components of a stressful environment include on-call responsibilities, high workload, competitive training, peer pressure, a large volume of content to study, and financial burdens [11]. A meta-analysis reported depression in approximately one-third of medical students globally [12], which may contribute to anxiety, burnout, suicidal thoughts, and substance abuse [11].

In a local context, a survey to assess the perceived stress and psychological well-being of 281 college students from 6 states in India showed a negative effect of perceived stress on their psychological well-being. A higher level of internet addiction negatively affected psychological well-being, whereas perceived social support from friends, family, and significant others was positively associated with well-being among college students [13]. Female students reported a higher level of well-being even though their stress levels were similar to male students [14].

In a study conducted by Jeyaraj et al [15] among college students in Maharashtra, India, stress was observed in 24.4% of students. Moderate to severe stress was experienced by 14.4% of students. Academic factors and lifestyle were significantly associated predictors of stress among college students. Another large survey study in India highlighted that more than 80% of medical students experienced burnout and were at higher risk of mental disorders and lower well-being [16]. This was also corroborated by additional studies that reported high psychological distress, burnout [17], perceived stress [18], poor well-being, stress, and depression in Indian medical students [19,20].

In India, mental health problems are prevalent in the young adult population, and since India is home to the world’s largest population of young people, it has become a matter of critical concern [21]. The World Health Organization reported that 8% of the Indian population has common mental disorders, of which 4.5% and 3.5% experience depression and anxiety, respectively [16].

However, conventional evidence-based psychological treatments have certain limitations associated with them, such as lack of access, stigma, and difficulty in affording the services [22]. Additionally, the limited availability of mental health professionals exacerbates the situation [23]. In this scenario, digital health interventions have the potential to address these challenges as they can reach a larger audience, are more cost-effective, and avoid the risk of stigma [24]. Targeted use of technology can be more effective in the health and well-being domain [25]. Various digital interventions, including computer-based [26,27], web-based [28,29], mobile-based (SMS text message–based and app-based), and virtual reality–based [30-32] interventions have been used for depression, anxiety, stress, sleep quality, psychological well-being, and mindfulness.

There is limited research available on digital health interventions in enhancing overall well-being among college students in India [33]. Further studies have shown that use of the health belief model (HBM) for student well-being is effective [34]. Hence, this study aims to explore factors affecting the well-being of students and develop and design a tailored, internet- and mobile-enabled digital health intervention based on the HBM to enhance well-being among college students in Indian settings.

### Study Objective

The objective of the study is to design, develop and pilot-test a digital health platform to enhance student well-being. The study aims are (1) to identify multiple determinants and factors impacting well-being among undergraduate and graduate students; (2) to design and develop a digital health intervention based on the HBM, incorporating the findings of aim 1 to address the well-being issues among undergraduate and graduate students; (3) to evaluate the feasibility and acceptance of the proposed digital health intervention; and (4) to evaluate the role of the digital health intervention, as well as the roles of adherence to and engagement with it, in enhancing the well-being of undergraduate and graduate students.

## Methods

### Study Design and Population

For aim 1, a cross-sectional study will be conducted to identify multiple determinants affecting the well-being of the students. Five thousand participants will be recruited across Gujarat and Tamil Nadu, India. Current undergraduates and graduates will be recruited from Parul University in Vadodara, Gujarat, as well as Panimalar Medical College Hospital and Research Institute, Panimalar Engineering College, Panimalar Institute of Technology, and Panimalar College of Nursing in Chennai, Tamil Nadu, using a convenience sampling approach. The participants will be recruited from their respective institutes with prior permission from their departments. The survey will be filled out by the students using their mobile devices or from the computer labs of the institutes. The survey will consist of validated tools, which are described in Multimedia Appendix 1 [35-37].

For aim 2, we will use a mixed methods approach and a quasi-experimental research design to gather data. Baseline data will be collected from aim 1 using predefined questionnaires and validated tools. Additionally, 3 focus group discussions (FGDs) will be conducted at each study site to identify the barriers that students face to manage their well-being. Two focus groups at each site is considered to be ideal [38]. Consenting participants from the baseline survey will be invited via an email to participate in the FGDs; they will then be given access to the mobile- and web-based platform to generate their initial user profiles. The data gathered from user profiles and FGDs will be used to formulate a student well-being index and also used to design and develop a tailored and context-specific digital health intervention (based on the principle of a human-centered approach) to address the well-being issues of the students. The quantitative data collected from the baseline survey and the qualitative factors will help in identifying causal factors in the environment of the students affecting their well-being [39]. The digital intervention will be based on a human-centered approach and will be designed after understanding an analysis of responses generated from the baseline survey and FGDs. The digital health intervention will be designed as per the needs identified from the FGDs and the baseline survey with population health informatics.

For aim 3 and aim 4, we will perform a randomized controlled trial. Based on the well-being index generated, high-risk individuals will be divided randomly into an intervention group and a control group. The digital health intervention will be a mobile- and web-based platform for students designed to provide tailored well-being–related education and awareness. A human-centered approach will be used to design and develop the intervention by incorporating the students’ needs and preferences to ensure an optimal technology-enabled intervention [40]. The study will also use the HBM in designing the digital health intervention, where HBM will be used as a framework to aid in the factors that influence implementation and the persistence of students in maintaining well-being. The HBM is one of the most widely used theoretical frameworks for understanding behavioral change. The model holds that an individual’s likelihood of adopting and maintaining a new behavior is heavily influenced by their perceptions of the health threat and the behavior being proposed to address that threat [41]. The intervention will include modules for the physical, social, and mental well-being of the students. The study participants will be able to access the personalized, human-centered digital health intervention through their computers or mobile phones depending on the technology platform available to them. The intervention will be tailored and will be delivered via interactive messages addressing the various dimensions of well-being among undergraduates and graduate students. Study participants in the control group will be provided with an educational booklet addressing health information related to student well-being. Pre- and postevaluation of the individuals in the intervention and control groups will be conducted to assess the impact of the digital health intervention. The feasibility, adherence, engagement, and acceptability of the system will be assessed.

### Study Eligibility

The eligible study participants will be aged 18 years or older; be enrolled in Parul University, Panimalar Medical College Hospital and Research Institute, Panimalar Engineering College, Panimalar Institute of Technology, or the Panimalar College Of Nursing; have access to a Wi-Fi or internet-enabled device; and consent to participate in the study. Participants younger than 18 years, not enrolled in these institutes, or not providing consent will be excluded from the study.

### Data Collection, Data Entry, and Quality Assurance

To ensure efficiency and high-quality data collection and processing, we will (1) use a well-trained team of field-workers, (2) use a clearly defined study manual, (3) conduct weekly meetings with the research team, and (4) maintain logs of all patient contacts. Confidentiality of the information obtained from the participants will be ensured. The information will be accessible to members of the research team only.

### Variable Assessment

#### Sociodemographic Profile

Data will be gathered from the participants on age, gender, income level, education level, region of residence, marital status, children, and religion. The socioeconomic status of the participants’ families will be evaluated with an updated version of the original Kuppuswamy Scale [42].

#### Health Status Profile

The height and weight of the participants will be measured to calculate BMI, as Sarwer and Polonsky [43] suggested that BMI has an impact on well-being. Any self-reported disability will be assessed. Predefined questionnaires will be used to gather the data.

#### Familiarity With Technology

We will gather data on access to desktop or laptop computers, tablets, and mobile phones, as well as the type of phone possessed, knowledge of texting, and access to and familiarity with the internet. Predefined questionnaires will be used to gather the data.

#### Health Behavior

Health behavior data will include the consumption of tobacco, use of alcohol, smoking, and sleep duration. Predefined questionnaires will be used to gather the data.

#### College Profile

Data will be collected on the residential status and prior education of the students (within the city or outside the city). Predefined questionnaires will be used to gather the data.

### Data Security and Privacy

Data security will be maintained by frequent backup in password-protected computers locked in the principal investigator’s office. The information will be accessed by the research team only. Locked data will be stored for 5 years from the point of study completion, at which time they will be destroyed [44].

### Outcomes

The study will help to identify multiple determinants affecting the students’ well-being. Additionally, it will explore the role of digital health interventions in enhancing good health and well-being among the students. The study will also help in designing and developing individualized, tailored, and interactive internet-enabled digital health interventions to manage the well-being of undergraduate and graduate students.

### Outcome Variables

The outcomes will be assessed with the tools described in this section.

#### General Well-Being

The General Well-Being Schedule will be used to gather data. The scale covers 6 dimensions, including anxiety, depression, general health, positive well-being, self-control, and vitality, to measure mental and physical well-being [45].

#### Perceived Social Support

The Multi-Dimensional Scale of Perceived Social Support will be used to collect the data. It includes subscales for support from family, friends, and significant others to assess perceived social support [46].

#### Mental Well-Being

Two components will be measured to assess mental wellness: stress and depression. Depression will be measured via the 8-item Patient Health Questionnaire [47] on a 4-point Likert scale that ranges from 0 (“not at all”) to 3 (“nearly every day”). Stress will be measured through the 10-item Perceived Stress Scale [48], which ranges from “never” to “very often” on a 5-point Likert scale.

#### Family Demands

Family demands are measured through a work-family conflict scale. It has 5 items on a Likert scale, from 1 (“strongly disagree”) to 7 (“strongly agree”) [49].

#### System Acceptability

System acceptability will be evaluated through the Client Satisfaction Questionnaire–8 to assess the acceptability of the digital health intervention [35].

#### General Self-Efficacy

General self-efficacy will be evaluated through the General Self-Efficacy Scale [36]. It is a 10-item scale to assess the ability to cope with daily challenging demands or situations in life.

#### Stigma

The Stigma Scale will be used to assess stigma. It has 42 statements on a 5-point Likert scale, ranging from “strongly agree” to “strongly disagree” [37].

#### Perceived Ease of Use

The Perceived Ease of Use questionnaire will be used to assess the feasibility of the digital health intervention [50].

#### Diet and Behavior Scale

A 29-item questionnaire called the Diet and Behavior Scale is used to gauge people’s intake of typical dietary components. The Diet and Behavior Scale’s first component focuses on the respondent’s typical frequency of consumption of common foods and beverages. The answers to the questions are given on a scale of 1 to 5, with 1 being “never,” 2 “once a month,” 3 “once or twice a week,” 4 “most days,” and 5 “every day.” The second segment is open ended and looks into the normal serving sizes for 11 popular foods and beverages [51].

#### Other Tools, Follow-Up, and Engagement

Tools other than the Perceived Ease of Use questionnaire and Client Satisfaction Questionnaire–8 will be used before and after the digital health intervention to assess its usefulness. Individuals will be followed up for a period of 3 months at regular intervals to assess adherence to the well-being modules provided in the digital health intervention. Engagement with the digital health intervention will be assessed by monitoring the number of weekly logins, completion of tasks in a timely manner, and submission of regular data.

### Data Analysis

The baseline data for generating the well-being profile include sociodemographic profile, college profile, health status profile, familiarity with technology, and health behavior. Outcome variables will be assessed using the General Well-Being Schedule, Multidimensional Scale of Perceived Social Support, Personal Health Questionnaire Depression Scale, Perceived Stress Scale, Work-Family Conflict Scale, Client Satisfaction Questionnaire–8, Perceived Ease of Use questionnaire, General Self-Efficacy Scale, Nutrition Behavior Scale, and Stigma Scale. A descriptive analysis will be carried out using the baseline data; also, cross tabulation of sociodemographic factors with variables gathered from the instruments will be done. Two FGDs will be conducted per site to assess the perception of students on how their well-being can be addressed. Thematic analysis will be done in accordance with AMEE Guide No. 131 for qualitative data collected through FGDs [52]. For continuous variables, means and SDs will be reported, and for categorical variables, frequencies will be calculated. All statistical analysis will be performed using SAS (version 9.3; SAS Institute), and results will be reported as *P* values and 95% CIs.

### Project Timeline and Milestones

A detailed study timeline is presented in Table 1.

**Table 1:** Scheduled timeline of tasks in the student well-being study.

Task	Months									
	1-2	3	4	5	6-7	8-10	11-13	14-15	16-19	20-24
Development of study protocol and ethical approval	✓									
Baseline data collection		✓	✓	✓						
Focus group discussions and development of intervention					✓	✓				
Deployment of the well-being intervention and recruitment of the target sample into intervention and control groups							✓			
Follow-up data collection							✓	✓		
Postdeployment assessment of intervention								✓		
Review of collected data by the research team								✓	✓	
Data analysis									✓	
Scientific publication										✓
Dissemination (report writing and presentations)										✓

### Ethical Considerations

The study, bearing protocol number PMCHRI-IHEC-071, gained approval from the Panimalar Medical College Hospital and Research Institute Institutional Human Ethics Committee (Central Drugs Standard Control Organization Registration; ECR/1399/Inst/TN/2020) in January 2021 (PMCH&RI/IHEC/2021/078, dated December 13, 2021). Results of the study will be disseminated through national and international conference presentations and peer-reviewed publications. Findings of the study will also be disseminated to local and state officials and policy makers for data-driven, evidence-based decision-making.

The institutional review board–approved informed consent form will be collected from the eligible study participants by the research team. The consent form will include information on the study, the duration of the study, and the benefits of the study results for the participants. Participants who are willing to participate will be enrolled in the study via email. Study participants can withdraw from the study at any time. All data, including withdrawals, will be reported in the final analysis. No monetary compensation will be given for participation in the study.

## Results

The proposed research is an unfunded study. The enrollment of the individuals in the study began in October 2022. Data gathered will be analyzed using SAS and the results will be reported as 95% CIs and *P* values. Thematic analysis will be done of the qualitative data from the FGDs. The proposed research study will help in identifying determinants of well-being among college students. It will also help in designing tailored, internet-based, interactive, digital health interventions to enhance student well-being.

## Discussion

The research project will help in assessing the factors affecting the well-being of undergraduate students. It will also aid in developing and implementing data-driven, human-centered, interactive interventions to improve overall well-being among undergraduate and graduate students. It will explore the role of digital health interventions in enhancing the good health and well-being of students. Additionally, considering the barriers related to delivery of digital interventions for mental health and the unique needs of Indian college students, it is imperative to fill the research gap and implement research studies to design, develop, and assess the usability, acceptability, and feasibility of digital interventions tailored to students’ needs [51,53]. The study will provide an outline of the well-being and also the potential academic stress of college students in India [54].

## Appendix

Multimedia Appendix 1 Full text of instruments used in the study.

## Abbreviations

FGD: focus group discussion
HBM: health belief model

## Data Availability

The data sets generated during and/or analyzed during the current study will be made available by the corresponding author on reasonable request.
