# Supplementary material for: Design, Develop, and Pilot-Test a Digital Platform to Enhance Student Well-Being: Protocol for a Mixed-Methods Study
Source: JMIR Res Protoc. 2024 Jul 23;13:e39779. doi: 10.2196/39779 (PMC11303900; doi:10.2196/39779)
Supplement: Multimedia Appendix 1 [file resprot_v13i1e39779_app1.docx]

**Multimedia Appendix 1.** Full text of the self-administered questionnaire.

1. **DATA COLLECTOR INFORMATION**

**A1. Date of visit** dd-mm-yyyy

**A2. Time of visit** _ _:_ _ hours

**A3. Names of Data Collection team**

**A4. Mode of travel (to and from the location)**

1. Vehicle provided by team/responsible organization
2. Public transport
3. Walk
4. Other, please specify_____
5. **GEOGRAPHICAL INFORMATION: The vehicle name of the Institution /University-**

**B2. Pin code-**

**B3. Location**

1. **INDIVIDUAL PROFILE**

**C1. Name of respondent:**

**C2. Email id:**

**C3.Phone number**

**C4. SOCIO-DEMOGRAPHIC**

**C4a. Age group (in years)**

1. 18-22
2. 23-27
3. 28-31
4. 32-36
5. 37-41
6. 41 and above

**C4b. Gender**

1. Male
2. Female
3. Other, please specify _________

**C4c. Education Level**

1. High school certification
2. Undergraduate
3. Graduate
4. Post graduate
5. Profession or Honours

**C4d. Marital Status**

1. Unmarried
2. Married
3. Divorced/Separated
4. Widowed

**C4e. Religion**

1. Hindu
2. Sikh
3. Christian
4. Muslim
5. Other, please specify___________
6. Not willing to tell

**C4f. Region of Residence**

1. Urban
2. Rural
3. Slum

**C4g. Do you have a child/children?**

1. Yes
2. No

C4ga. If yes, number of children, please specify _________

C4gb. Age of each child, please specify _________

**Socio-economic status:** Source: https://pdfs.semanticscholar.org/c906/10dcdc166379ddab9859eb0c0dbb56e9394e.pdf

| **Table 1:** Occupation of the Head of the family | | |
| --- | --- | --- |
| **S. No.** | **Occupation of the Head** | **Score** |
| 1 | Legislators, Senior Officials & Managers | 10 |
| 2 | Professionals | 9 |
| 3 | Technicians and Associate Professionals | 8 |
| 4 | Clerks | 7 |
| 5 | Skilled Workers and Shop & Market Sales Workers | 6 |
| 6 | Skilled Agricultural & Fishery Workers | 5 |
| 7 | Craft & Related Trade Workers | 4 |
| 8 | Plant & Machine Operators and Assemblers | 3 |
| 9 | Elementary Occupation | 2 |
| 10 | Unemployed | 1 |
| **Table 2:** Education of the Head of the family | | |
| **S. No.** | **Education of the Head** | **Score** |
| 1 | Profession or Honours | 7 |
| 2 | Graduate | 6 |
| 3 | Intermediate or diploma | 5 |
| 4 | High school certificate | 4 |
| 5 | Middle school certificate | 3 |
| 6 | Primary school certificate | 2 |
| 7 | Illiterate | 1 |

**Table 3**: Total monthly income of the family

| S.no | Updated Monthly  Family Income in  Rupees (2012) | Updated Monthly  Family Income in  Rupees (2018) | Updated Monthly  Family Income in  Rupees (2020) | Updated Monthly  Family Income in  Rupees (2021) | Score |
| --- | --- | --- | --- | --- | --- |
| 1 | ≥ 30,375 | ≥ 126,360 | ≥ 199,862 | ≥123,322 | 12 |
| 2 | 15,188–30,374 | 63,182–126,359 | 99,931–199,861 | 61,663-123,321 | 10 |
| 3 | 11,362–15,187 | 47,266–63,181 | 74,755 –99,930 | 46129-61,662 | 6 |
| 4 | 7594–11,361 | 31,591–47,265 | 49,962–74,755 | 30,831-46,128 | 4 |
| 5 | 4556–7593 | 18,953–31,590 | 29,973– 49,961 | 18,497-30,830 | 3 |
| 6 | 1521–4555 | 6327–18,952 | 10,002–29,972 | 6,175-18,496 | 2 |
| 7 | ≤ 1520 | ≤ 6326 | ≤ 10,001 | ≤ 6174 | 1 |

1. **HEALTH STATUS PROFILE**

**D1. Do you have any underlying physician confirmed illnesses?**

1. Yes
2. No
3. I do not know

D1a. If yes, please specify________

**Do you have any physician confirmed disability?**

**D4. Height (in cm)**

**D5. Weight (in kg)**

**D6. Body mass index (BMI) (kg/m2)**

**E.ACCESS AND USE OF INFORMATION AND COMMUNICATION TECHNOLOGIES**

**E1. Do you own any of the following devices?**

**E1a. Desktop computer**

a.Yes

b.No

**E1b.Laptop**

a.Yes

b.No

**E1c.Tablet device**

a.Yes

b.No

**E1d.Mobile phone**

a.Yes

b.No

**E1da.** If yes, type of mobile phone please specify**_________**

**E2. Do you have access to Internet?**

a.Yes

b.No

**E2a.** If yes, where do you access the internet**?**

a.Home

b.Campus

c.Cybercafe

d.Hostel

E3a. Do you have knowledge of texting /messaging?

a.Yes

b.No

c. Not willing to tell

**F.HEALTH BEHAVIOUR**

**F1. Do you smoke cigarettes?**

a.Yes

b. No

c. Not willing to tell

**F1a.** If yes, how many cigarettes in a day

**F2. Do you consume tobacco?**

a.Yes

b. No

c. Not willing to tell

**F2a.** If Yes, How many packets in a day

**F3.Do you consume alcohol?**

a.Yes

b.No

c.Not willing to tell

**F3a**. If yes, how many ml in a day

F4. Consumption of any other substance

- - - 1. Yes
      2. No
      3. Not willing to tell

F4a. If yes, please specify the quantity and frequency.

**F4.** Sleep profile

**a) On an average week night (that is, Monday through Friday), how many hours of sleep do you get? Please give your answer in hours and minutes.**

Hours

Minutes

**b) In the past 30 days, how would you rate your sleep quality overall?**

○Very good

○Fairly good

○Fairly bad

○Very bad

○Don’t know/Not Sure

**G College profile**

**G1. Do you live in hostel?**

a)Yes

b) No

**G2. Do you live in a PG (paying guest)?**

**a**)Yes

b) No

**G3. Do you live in a residence?**

**a**)Yes

b) No

**G4.Have you ever lived in the hostel before?**

a)Yes

b) No

**G5. Have you ever lived in the PG before?**

a)Yes

b) No

**G6.Was you prior education within the city**

a)Yes

b) No

**G7**.**Was you prior education outside the city**

a)Yes

b) No

**G7**.**Was you prior education within the state**

a)Yes

b) No

**G7**.**Was you prior education outside the state**

a)Yes

b) No

**D Instruments**

1. **General well-being schedule 18 items:** Source: https://cde.nlm.nih.gov/formView?tinyId=YkER84OKU
2. **How have you been feeling in general? (DURING THE PAST MONTH)**

 In excellent spirits

 In very good spirits

 In good spirits mostly

 I've been up and down in spirits a lot

 In low spirits mostly

 In very low spirits

1. **Have you been bothered by nervousness or your nerves? (DURING THE PAST MONTH)**

 Extremely so -- to the point where I could not work or take care of things

 Very much so

 Quite a bit

 Some -- enough to bother me

 A little

 Not at all

1. **Have you been in firm control of your behaviour, thoughts, emotions, or feelings? (DURING THE PAST MONTH)**

 Yes, definitely so

 Yes, for the most part

 Generally so

 Not too well

 No, and I am somewhat disturbed

 No, and I am very disturbed

1. **Have you felt so sad, discourages, hopeless, or had so many problems that you wondered if anything was worthwhile? (DURING THE PAST MONTH)**

 Extremely so -- to the point that I have just about given up

 Very much so

 Quite a bit

 Some -- enough to bother me

 A little bit

 Not at all

1. **Have you been under or felt you were under any strain, stress, or pressure? (DURING THE PAST MONTH)**

 Yes -- almost more than I could bear or stand

 Yes -- quite a bit of pressure

 Yes -- some - more than usual

 Yes -- some - but about usual

 Yes - a little

 Not at all

1. **How happy, satisfied, or pleased have you been with your personal life? (DURING THE PAST MONTH)**

 Extremely happy -- could not have been more satisfied or pleased

 Very happy

 Fairly happy

 Satisfied -- pleased

 Somewhat dissatisfied

 Very dissatisfied

1. **Have you had any reason to wonder if you were losing your mind, or losing control over the way you act, talk, think, feel, or of your memory? (DURING THE PAST MONTH)**

 Not at all

 Only a little

 Some -- but not enough to be concerned or worried about

 Some and I have been a little concerned

 Some and I am quite concerned

 Yes, very much so and I am very concerned

1. **Have you been anxious, worried, or upset? (DURING THE PAST MONTH)**

 Extremely so -- to the point of being sick, or almost sick

 Very much so

 Quite a bit

 Some -- enough to bother me

 A little bit

 Not at all

1. **Have you been waking up fresh and rested? (DURING THE PAST MONTH)**

 Every day

 Most every day

 Fairly often

 Less than half the time

 Rarely

 None of the time

1. **Have you been bothered by any illness, bodily disorder, pains, or fears about your health? (DURING THE PAST MONTH)**

 All of the time

 Most of the time

 A good bit of the time

 Some of the time

 A little of the time

 None of the time

1. **Has your daily life been full of things that were interesting to you? (DURING THE PAST MONTH)**

 All of the time

 Most of the time

 A good bit of the time

 Some of the time

 A little of the time

 None of the time

1. **Have you felt down hearted and blue? (DURING THE PAST MONTH)**

 All of the time

 Most of the time

 A good bit of the time

 Some of the time

 A little of the time

 None of the time

1. **Have you been feeling emotionally stable and sure of yourself? (DURING THE PAST MONTH)**

 All of the time

 Most of the time

 A good bit of the time

 Some of the time

 A little of the time

 None of the time

1. **Have you felt tired, worn out, used-up, or exhausted? (DURING THE PAST MONTH)**

 All of the time

 Most of the time

 A good bit of the time

 Some of the time

 A little of the time

 None of the time

1. **How concerned or worried about your HEALTH have you been? (DURING THE PAST MONTH)**

 0 - Not concerned at all

 1

 2

 3

 4

 5

 6

 7

 8

 9

 10 - Very concerned

1. **How RELEAXED or TENSE have you been? (DURING THE PAST MONTH)**

 0 - Very relaxed

 1

 2

 3

 4

 5

 6

 7

 8

 9

 10 - Very tense

1. **How much ENERGY, PEP, and VITALITY have you felt? (DURING THE PAST MONTH)**

 0 - No energy at all, listless

 1

 2

 3

 4

 5

 6

 7

 8

 9

 10 - Very energetic, dynamic

1. **How DEPRESSED or CHEERFUL have you been? (DURING THE PAST MONTH)**

 0 - Very depressed

 1

 2

 3

 4

 5

 6

 7

 8

 9

 10 - Very cheerful

**2) Multidimensional Scale of Perceived Social Support**: Source: https://www.tnaap.org/documents/mspss-multidimensional-scale-of-perceived-social.

Instructions: We are interested in how you feel about the following statements. Read each statement carefully. Indicate how you feel about each statement.

Circle the “1” if you Very Strongly Disagree

Circle the “2” if you Strongly Disagree

Circle the “3” if you Mildly Disagree

Circle the “4” if you are Neutral

Circle the “5” if you Mildly Agree

Circle the “6” if you Strongly Agree

Circle the “7” if you Very Strongly Agree

| 1. | There is a special person who is around when I am in need. | 1 | 2 | 3 | 4 | 5 | 6 | 7 | SO |
| --- | --- | --- | --- | --- | --- | --- | --- | --- | --- |
| 2. | There is a special person with whom I can share my joys and sorrows. | 1 | 2 | 3 | 4 | 5 | 6 | 7 | SO |
| 3. | My family really tries to help me. | 1 | 2 | 3 | 4 | 5 | 6 | 7 | Fam |
| 4. | I get the emotional help and support I need from my family. | 1 | 2 | 3 | 4 | 5 | 6 | 7 | Fam |
| 5. | I have a special person who is a real source of comfort to me. | 1 | 2 | 3 | 4 | 5 | 6 | 7 | SO |
| 6. | My friends really try to help me. | 1 | 2 | 3 | 4 | 5 | 6 | 7 | Fri |
| 7. | I can count on my friends when things go wrong. | 1 | 2 | 3 | 4 | 5 | 6 | 7 | Fri |
| 8. | I can talk about my problems with my family. | 1 | 2 | 3 | 4 | 5 | 6 | 7 | Fam |
| 9. | I have friends with whom I can share my joys and sorrows. | 1 | 2 | 3 | 4 | 5 | 6 | 7 | Fri |
| 10. | There is a special person in my life who cares about my feelings. | 1 | 2 | 3 | 4 | 5 | 6 | 7 | SO |
| 11. | My family is willing to help me make decisions. | 1 | 2 | 3 | 4 | 5 | 6 | 7 | Fam |
| 12. | I can talk about my problems with my friends. | 1 | 2 | 3 | 4 | 5 | 6 | 7 | Fri |

The items tended to divide into factor groups relating to the source of the social support, namely family (Fam), friends (Fri) or significant other (SO).

**3. Personal Health Questionnaire Depression Scale (PHQ-8):** Source: <https://selfmanagementresource.com/wp-content/uploads/2019/07/English_-_phq.pdf>

Over the **last 2 weeks**, how often have you been bothered by any of the following problems?

(Circle one number on each line)

| **How often during the past 2**  **weeks were you bothered by...** | **Not**  **at all** | **Several**  **days** | **More than half**  **the days** | **Nearly**  **every day** |
| --- | --- | --- | --- | --- |
| Little interest or pleasure in  doing things | 0 | 1 | 2 | 3 |
| Feeling down, depressed, or hopeless | 0 | 1 | 2 | 3 |
| Trouble falling or staying asleep, or sleeping too much | 0 | 1 | 2 | 3 |
| Feeling tired or having little energy | 0 | 1 | 2 | 3 |
| Poor appetite or overeating | 0 | 1 | 2 | 3 |
| Feeling bad about yourself, or that you are a failure, or have let yourself or your family down | 0 | 1 | 2 | 3 |
| Trouble concentrating on things, such as reading the newspaper or watching television | 0 | 1 | 2 | 3 |
| Moving or speaking so slowly that other people could have noticed. Or the opposite being so fidgety or restless that you have been moving around a lot more than usual | 0 | 1 | 2 | 3 |

**4**. **Perceived stress scale**: Source <https://www.das.nh.gov/wellness/docs/percieved%20stress%20scale.pdf>

**For each question choose from the following alternatives**

**0-never 1-almost never 2 –sometimes 3-fairly often 4-very often**

In the last month, how often have you been upset because of something that happened unexpectedly?

In the last month, how often have you felt that you were unable to control the important things in your life?

In the last month, how often have you felt nervous and stressed?

In the last month, how often have you felt confident about your ability to handle?

your personal problems?

In the last month, how often have you felt that things were going your way?

In the last month, how often have you found that you could not cope with all the things that you had to do?

In the last month, how often have you been able to control irritations in your life?

In the last month, how often have you felt that you were on top of things?

In the last month, how often have you been angered because of things that happened that were outside of your control?

In the last month, how often have you felt difficulties were piling up so high that

you could not overcome them?

**5. Work-family conflict scale:** Source**:** <https://alswh.org.au/wp-content/uploads/2020/08/DSSSection2.7WorkFamilyConflictScale.pdf>

For the following scale please rate how much you agree with the following statements by circling the appropriate number.

1. Very strongly disagree
2. Strongly disagree
3. Disagree
4. Neither agree nor disagree
5. Agree
6. Strongly agree
7. Very strongly agree

|  | Very strongly disagree | Strongly disagree | Disagree | Neither agree nor disagree | Agree | Strongly agree | Very strongly agree |
| --- | --- | --- | --- | --- | --- | --- | --- |
| The demands of my work interfere with my home and family life | 1 | 2 | 3 | 4 | 5 | 6 | 7 |
| The amount of time my job takes up makes it difficult to fulfill family responsibilities | 1 | 2 | 3 | 4 | 5 | 6 | 7 |
| Things I want to do at home do not get done because of the demands my job puts on me | 1 | 2 | 3 | 4 | 5 | 6 | 7 |
| My job produces strain that makes it difficult to fulfil family duties | 1 | 2 | 3 | 4 | 5 | 6 | 7 |
| Due to work-related duties, I have to make changes to my plans for family activities | 1 | 2 | 3 | 4 | 5 | 6 | 7 |

**7. Tool for Acceptance: CSQ-8 Source:** Larsen DL, Attkisson CC, Hargreaves WA, Nguyen TD. Assessment of client/patient satisfaction: development of a general scale. Evaluation and program planning. 1979 Jan 1;2(3):197-207.

Instructions for participants:

Please help us improve our service by answering some questions about the help that you have received.

We are interested in your honest opinions, whether they are positive or negative. Please answer all of the questions. We also welcome your comments and suggestions. Thank you very much. We appreciate your help.

1. How would you rate the quality of program you received?

Excellent (4)

Good (3)

Fair (2)

Poor (1)

2. Did you get the kind of program you wanted?

No, definitely not (1)

No, not really (2)

Yes, generally (3)

Yes, definitely (4)

3. To what extent has our program met your needs?

Almost all of my needs have been met (4)

Most of my needs have been met (3)

Only a few of my needs have been met (2)

None of my needs have been met (1)

4. If a friend were in need of similar help, would you recommend our program to him or her?

No, definitely not (1)

No, I don’t think so (2)

Yes, I think so (3)

Yes, definitely (4)

5. How satisfied are you with the amount of help you received?

Quite dissatisfied (1)

Indifferent or mildly dissatisfied (2)

Mostly satisfied (3)

Very satisfied (4)

6. Have the program you received helped you to deal more effectively with your problems?

Yes, they helped a great deal (4)

Yes, they helped somewhat (3)

No, they really didn’t help (2)

No, they seemed to make things worse (1)

7. In an overall, general sense, how satisfied are you with the program you received?

Very satisfied (4)

Mostly satisfied (3)

Indifferent or mildly dissatisfied (2)

Quite dissatisfied (1)

8. If you were to seek help again, would you come back to our program?

No, definitely not (1)

No, I don’t think so (2)

Yes, I think so (3)

Yes, definitely (4)

**Perceived ease of use (PEOU) questionnaire items**

| **Variables/items** | **Questions asked** | **strongly disagree (1)** | **disagree (2)** | **Neutral (3)** | **Agree (4)** | **Strongly agree (5)** |
| --- | --- | --- | --- | --- | --- | --- |
| Understandable and clear interaction | Knowing about health through the use of the digital health Intervention |  |  |  |  |  |
| Flexible interaction | Did you find the digital health intervention easy to navigate? |  |  |  |  |  |
| Easy usage | Did you find the digital health intervention easy to use |  |  |  |  |  |
| General easiness | Overall, did you find the digital health intervention easy |  |  |  |  |  |
| Easy learning | Was it easy to learn about your health through the digital health intervention |  |  |  |  |  |
| Skill enhancement | Did you find that your knowledge/skill has enhanced through the digital health intervention to maintain a better health/lifestyle |  |  |  |  |  |
| Understandable and clear interaction | Knowing about health through the use of the digital health intervention was clear and understandable |  |  |  |  |  |

**8. General Self efficacy scale:** Source: Schwarzer R & Jerusalem M. Generalized self-efﬁcacy scale. In J Weinman, S Wright, & M Johnston. Measures in health psychology:

A user’s portfolio. Causal and control beliefs. Windsor, England:

NFER-NELSON; 1995: 35-37.

https://pcna.net/wp-content/uploads/2018/12/16e_the_general_self_efficacy_scale.pdf

16E

The following scale was developed to evaluate the coping ability of daily living

Response format

1= Not at all true 3= Moderately true

2= hardly true 4= exactly true

| Statement | Write the number that best describes your opinion |
| --- | --- |
| I can always manage to solve difﬁcult problems if I try hard enough. |  |
| If someone opposes me, I can ﬁnd the means and ways  to get what I want |  |
| It is easy for me to stick to my aims and accomplish  my goals. |  |
| I am conﬁdent that I could deal efﬁciently with  unexpected events. |  |
| Thanks to my resourcefulness, I know how to handle  unforeseen situations |  |
| I can solve most problems if I invest the necessary effort. |  |
| I can remain calm when facing difﬁculties because I can rely on my coping abilities. |  |
| When I am confronted with a problem, I can usually ﬁnd several solutions. |  |
| If I am in trouble, I can usually think of a solution. |  |
| I can usually handle whatever comes my way |  |

Add up the numbers from each row in the last column. This total equals your self-efﬁcacy score. The higher the score, the greater your self-efﬁcacy or conﬁdence in your ability to successfully manage an illness or follow through with behavior change. This score may change over time.

Adapted from

Schwarzer R & Jerusalem M. Generalized self-efﬁcacy scale.

In J Weinman, S Wright, & M Johnston. Measures in health psychology:

A user’s portfolio. Causal and control beliefs. Windsor, England:

NFER-NELSON; 1995: 35-37.

**9. Stigma scale: Source:** King M, Dinos S, Shaw J, Watson R, Stevens S, Passetti F, Weich S, Serfaty M. The Stigma Scale: development of a standardised measure of the stigma of mental illness. The British Journal of Psychiatry. 2007 Mar;190(3):248-54.

Each question scored 0 - 4 in the direction of greater stigma: A, scored 0 - 4 in direction of agreement; 1., scored 0 - 4 in direction of agreement; D, scored 0 - 4 in direction of disagreement.

| Statement | Strongly agree | Agree | Neither agree or disagree | Disagree | Strongly disagree |
| --- | --- | --- | --- | --- | --- |
| The general public is understanding of people with mental health problems (D) |  |  |  |  |  |
| Other people have made me feel ashamed of myself because of my mental health  problems (A)  0.38 |  |  |  |  |  |
| The way people have treated me upsets me (A) |  |  |  |  |  |
| I have been discriminated against by housing departments/landlords because of my mental  health problems (A) |  |  |  |  |  |
| I have been discriminated against in education because of my mental health problems (A) |  |  |  |  |  |
| Sometimes I feel that I am being talked down to because of my mental health problems (A) |  |  |  |  |  |
| Having had mental health problems has made me a more understanding person (D) |  |  |  |  |  |
| I am to blame for my mental health problems (A) |  |  |  |  |  |
| I feel ashamed of myself that I have had mental health problems (A) |  |  |  |  |  |
| I do not feel bad about having had mental health problems (D) |  |  |  |  |  |
| Other people think less of me because I have had mental health problems (A) 11 Other people think less of me because I have had mental health problems (A) |  |  |  |  |  |
| Newspapers/television take a balanced view about mental health problems (D) |  |  |  |  |  |
| I am open to my family about my mental health problems (D) |  |  |  |  |  |
| I worry about telling people I receive psychological treatment (A) |  |  |  |  |  |
| Some people with mental health problems are dangerous (A) |  |  |  |  |  |
| Other people have never made me feel embarrassed because of my mental health  problems (D) |  |  |  |  |  |
| People have been understanding of my mental health problems (D) |  |  |  |  |  |
| I have been discriminated against by police because of my mental health problems (A) |  |  |  |  |  |
| I have been discriminated against by employers because of my mental health problems (A) |  |  |  |  |  |
| I have been physically threatened or attacked because of my mental health problems (A) |  |  |  |  |  |
| My mental health problems have made me more accepting of other people (D) |  |  |  |  |  |
| Very often I feel alone because of my mental health problems (A |  |  |  |  |  |
| I am scared of how other people will react if they find out about my mental health |  |  |  |  |  |
| I would have had better chances in life if I had not had mental health problems (A) |  |  |  |  |  |
| I would have had better chances in life if I had not had mental health problems (A) |  |  |  |  |  |
| I am as good as other people, even though I have had mental health problems (D) |  |  |  |  |  |
| I am as good as other people, even though I have had mental health problems (D) |  |  |  |  |  |
| I do not mind people in my neighbourhood knowing I have had mental health  problems (D) |  |  |  |  |  |
| I would say I have had mental health problems if I was applying for a job (D) |  |  |  |  |  |
| I worry about telling people that I take medicines/tablets for mental health problems (A) |  |  |  |  |  |
| People’s reactions to my mental health problems make me keep myself to myself (A) |  |  |  |  |  |
| I am angry with the way people have reacted to my mental health problems (A) |  |  |  |  |  |
| I have not had any trouble from people because of my mental health problems (D) |  |  |  |  |  |
| I have been discriminated against by health professionals because of my mental health  problems (A) |  |  |  |  |  |
| 33 People have avoided me because of my mental health problems (A) |  |  |  |  |  |
| People have insulted me because of my mental health problems (A) |  |  |  |  |  |
| Having had mental health problems has made me a stronger person (D) |  |  |  |  |  |
| I do not feel embarrassed because of my mental health problems (D) |  |  |  |  |  |
| I avoid telling people about my mental health problems (A) |  |  |  |  |  |
| Having had mental health problems makes me feel that life is unfair (A) |  |  |  |  |  |
| When I see or read something about mental health in the papers or television,  it makes me feel bad about myself (A) |  |  |  |  |  |
| I feel the need to hide my mental health problems from my friends (A) |  |  |  |  |  |
| I find it hard telling people I have mental health problems (A) |  |  |  |  |  |
| I do not understand the diagnosis I have been given (A) |  |  |  |  |  |

**Diet and Behaviour Scale (DABS)**

**Source:** [**https://orca.cardiff.ac.uk/id/eprint/72317/2/ASmith%20-%20Food.pdf**](https://orca.cardiff.ac.uk/id/eprint/72317/2/ASmith%20-%20Food.pdf)

Section-I: Kindly rate the questions from Q1 to Q8 within a five-point scale (1 = never, 2 = once a month, 3 = once or twice a week, 4 = most days, 5 = every day

Q1. How often did you eat breakfast?

Q2. How often did you eat chocolate?

Q3. How often did you eat crisps?

Q4. How often did you eat 5 fruit or vegetables?

Q5. How often did you drink coffee?

Q6. How often did you drink tea?

Q7. How often did you drink cola?

Q8. How often did you drink energy drinks?

Q9. How often did you chew gum?

Q10. How often did you eat sweets?

Q11. How often did you eat fast-food?

Q12. How often did you eat an Indian or Chinese take-away?

Q13. How often did you eat pies or pasties?

Q14. How often did you eat processed meat?

Q15. How often did you eat fried fish?

Q16. How often did you eat oily fish?

Q17. How often did you eat chips?

Q18. How often did you eat beans of peas?

Section-II: From Q19 to Q29. Kindly mention the frequency of the food consumed

Q19. Cans of energy drinks per week

Q20. Cans of cola per week

Q21. Cups of coffee per week

Q22. Cups of tea per week

Q23. Packets of crisps per week

Q24. Bars of chocolate per week 2009

Q25. Burgers/hot dogs per week

Q26. Packs of chewing gum per week

Q27. Pieces of fruit per day

Q28. Portions of vegetables per day

Q29. Pints of water per day
